# *Gardnerella* biofilm formation *in vitro* is facilitated by braided sutures: implications for cervical cerclage

**DOI:** 10.3389/fcimb.2026.1763531

**Published:** 2026-03-24

**Authors:** Hillary Zhou, Sudeshna Saha, Sydney Morrill, Thomas Kelly, Warren G. Lewis, Amanda L. Lewis

**Affiliations:** 1 Department of Obstetrics, Gynecology, and Reproductive Sciences, University of California San Diego (UCSD), La Jolla, CA, United States; 2 Department of Pediatrics, University of California San Diego (UCSD), La Jolla, CA, United States

**Keywords:** bacterial vaginosis (BV), biofilm, cervical cerclage, *Gardnerella*, suture

## Abstract

**Introduction:**

In pregnant individuals with certain indications, sutures may be placed circumferentially around the uterine cervix to prevent dilation. Compared to monofilament sutures, the use of braided suture materials has been linked with the development of a dysbiotic vaginal microbiome, as well as higher rates of infection-associated pregnancy outcomes such as chorioamnionitis and preterm birth. In bacterial vaginosis (BV) anaerobic bacteria, including pathogens, overgrow, forming biofilms in direct proximity to the host epithelium. Gardnerella is highly represented among bacterial vaginosis-like microbiotas.

**Methods:**

To test our working hypothesis that braided sutures may better support the establishment of high biomass bacterial biofilms compared to monofilament sutures, we measured the extent of Gardnerella bacteria biofilm formation on braided and monofilament sutures in the laboratory. Multiple Gardnerella strains were grown in the presence of braided or monofilament suture materials (polyester or polybutylate-coated polyester versus polypropylene or nylon), and the (biofilm) biomass was measured using crystal violet staining.

**Results:**

Sutures incubated without Gardnerella were included as controls. To compare staining of biofilm biomass between groups, one-way ANOVA was performed and Šidák was used for pairwise comparisons to control for multiple comparisons between groups. Gardnerella formed significantly more biofilm biomass (>10-fold) on braided polyethylene terephthalate (polyester) sutures compared to monofilament (polypropylene or nylon) sutures (p < 0.0001). This feature was applicable to multiple strains across different taxonomic subsets of Gardnerella.

**Discussion:**

Together with existing literature, these findings suggest that braided sutures might promote the development of dysbiotic BV-like microbiomes after cerclage placement by facilitating Gardnerella biofilm formation.

## Introduction

Cervical cerclage is a procedure often performed transvaginally with the goal of preventing a previable loss or preterm delivery. Surgical techniques and suture materials are varied at the discretion of the provider. In a McDonald procedure, a nonabsorbable suture is placed circumferentially around the cervix during pregnancy, with portions of the material and knot exposed to the vagina. Previously stated indications for cerclage have included cervical insufficiency, prior preterm birth(s), or shortened cervical length by ultrasound (Care et al., 2022). Clinical research has investigated certain aspects of cerclage, comparing the procedure to alternative treatments (Care et al., 2022), or comparing the use of specific suture materials (Berghella et al., 2012; Feng et al., 2023; Hodgetts Morton et al., 2024). In particular, the use of braided cerclage sutures has recently become a topic of interest, as different studies have suggested this material is associated with higher rates of poor pregnancy outcomes such as miscarriage, preterm birth (PTB), and chorioamnionitis (Kindinger et al., 2016; Hodgetts Morton et al., 2022; Hodgetts Morton et al., 2024). The exact methods and conclusions of the existing studies differ in important ways. While the earlier Kindinger study noted higher rates of PTB in braided versus monofilament suture use, a larger randomized controlled trial did not observe the same outcomes when comparing braided versus monofilament sutures. This study instead found other infection/inflammation-related maternal outcomes of chorioamnionitis and sepsis were associated with the use of braided sutures. However, specific outcomes have not been consistently linked with the microbiome or the use of braided sutures, and mechanisms of such relationships have not been studied using laboratory methods.

Bacterial vaginosis (BV) is a common condition of the vaginal microbiota characterized by a low number of healthy *Lactobacillus* and large numbers of *Gardnerella*, *Prevotella*, and other diverse anaerobes (Hillier, 1993; Fredricks et al., 2005; Ravel et al., 2011; Srinivasan et al., 2012). The etiology of BV is believed to rely on bacterial biofilms that form on the vaginal epithelium (Muzny et al., 2020; Muzny and Sobel, 2022; Sousa et al., 2023; Swidsinski et al., 2023). One of the criteria for clinical BV diagnosis is the presence of clue cells (i.e. vaginal epithelial cells studded with bacteria) in wet mount microscopy (Gardner and Dukes, 1955; Amsel et al., 1983; Srinivasan et al., 2012). Fluorescent *in situ* hybridization of vaginal biopsy specimens from individuals with BV have further revealed sheets of *Gardnerella* covering the epithelium (Swidsinski et al., 2005; Swidsinski et al., 2014).

In pregnancy, BV-like microbiomes and BV-associated bacteria have been linked with poor outcomes including amniotic fluid infections (Silver et al., 1989; Hitti et al., 2001; DiGiulio, 2012), chorioamnionitis (Hillier et al., 1988; Zhang et al., 2002; Rezeberga et al., 2008; Zhang et al., 2018), maternal bacteremia (Boggess et al., 1996), late miscarriage (Hay, 2004), and preterm birth (Silver et al., 1989; Boggess et al., 1996; DiGiulio, 2012). Clindamycin or metronidazole are common treatments for BV (Ferris et al., 1995; Abbe and Mitchell, 2023). However, while immediate effects of treatment are often positive, the recurrence of BV is substantial (more than half of those treated in some studies) (Bradshaw et al., 2006; Faught and Reyes, 2019), complicating remediation efforts aimed at improving health outcomes. Indeed, since there has not been a consistent improvement in pregnancy outcomes, screening and treatment for (asymptomatic) BV in pregnancy are not recommended by the US Preventive Services Task Force (Lewis and Laurent, 2020). However, a promising new protocol for partner treatment strongly reduced the incidence of BV recurrence (Vodstrcil et al., 2025). This study was not performed in pregnancy; however, it further supports the interpretation that treatments for BV are not fully effective due to our misunderstandings of the condition. This stands in contrast to a different interpretation: that the health outcomes associated with BV derive no benefit from its successful treatment. Biofilms are often credited as an explanation for BV pathophysiology and recurrence. However, the role of BV-associated biofilms on abiotic surfaces, and their relationship to maternal-fetal medicine, have not been directly pursued.

In a previous clinical study, participants underwent an indicated cervical cerclage and were randomized to receive either braided Mersilene (n=25) or monofilament Ethilon (n=24) sutures (Kindinger et al., 2016). To examine how suture types interact with the vaginal microbiome, the composition of vaginal bacteria was assessed before cerclage placement, and subsequently re-evaluated at 4- and 12-weeks post procedure. Individuals in the monofilament group had relatively stable vaginal microbiotas. However, those who received braided sutures disproportionately experienced dysbiotic BV-like changes in the microbiome, characterized by overgrowth of diverse anaerobic bacteria and low levels of *Lactobacilllus* during the first 4 weeks. The percent of participants in the braided suture group with BV-like vaginal microbiomes increased from 13% to 45%. The dysbiotic microbiome persisted in these participants for 16 weeks post-cerclage and was associated with pro-inflammatory markers and premature cervical vascularization (Kindinger et al., 2016), both features used as predictive markers for spontaneous preterm birth (Romero et al., 1989; Wei et al., 2010). This study provides important insights linking suture types used in cervical cerclage with changes in the vaginal microbiota. The authors suggested that the mechanism may relate to biofilm formation on the sutures, finding that vaginal bacteria such as *Escherichia coli* and *Lactobacillus jensenii* formed biofilms more robustly on braided compared to monofilament sutures (Gilbert and Lewis, 2019). However, neither of these species are associated with BV or BV-biofilms.

*Gardnerella* is one of the most abundant members of the BV-microbiome (Kindinger et al., 2016; Zhang et al., 2018). While not believed to be the sole cause of BV, *Gardnerella* can experimentally recreate some BV-like phenotypes in animal models (Morrill et al., 2020). For example, vaginal introduction of *Gardnerella* in mice leads to the appearance of clue-like cells (Gilbert et al., 2013), vaginal mucus degradation (Lewis et al., 2013), and cervical remodeling in pregnancy (Sierra et al., 2018), and appears to make other bacterial pathogens more likely to cause disease (Gilbert and Lewis, 2019; Gilbert et al., 2019; Morrill et al., 2020; Gilbert et al., 2021). This includes placental infections by vaginally introduced bacteria like beta hemolytic *Streptococcus (*Gilbert et al., 2021*).* Thus, the current study aimes to investigate the ability of *Gardnerella to* form biofilms on commonly used braided (polyethylene terephthalate) and monofilament (polypropylene and nylon) suture materials. We developed a new on-suture biofilm assay to test the hypothesis that *Gardnerella* would form more robust biofilms on braided compared to monofilament sutures. Our study includes multiple *Gardnerella* strains and a head-to-head comparison of the Mersilene and Ethilon sutures used in the aforementioned clinical study.

## Materials and methods

### Strains and culture conditions

Bacterial strains used in the assay include *Gardnerella* ATCC14019, JCP8151B, JCP8070, JCP8108, and JCP7672. *Gardnerella* ATCC14019 was ordered from the American Type Culture Collection. The remaining strains were isolated earlier from clinical specimens collected from the Washington University Contraceptive CHOICE Project, as previously described (Lewis et al., 2013). *Gardnerella* strains were cultivated using NYCIII agar and broth media. *Gardnerella* was selected because it is highly represented among bacterial vaginosis-like microbiotas, has been shown to encourage virulence of other urogenital pathogens, and is known to generate robust biofilms on plate wells in the laboratory and on the vaginal epithelium.

### Growth of Gardnerella biofilms on well plates

In a vinyl anaerobic chamber with 5% hydrogen gas, *Gardnerella* was streaked onto NYCIII agar and incubated anaerobically at 37 °C for 24 hours (hrs). 10 colonies of *Gardnerella* were then inoculated into NYCIII media and incubated anaerobically at 37 °C for 16 hrs (overnight). The optical density (OD_600_) of the overnight culture was measured in a spectrophotometer and diluted to an OD_600_ of 0.1 with fresh anaerobic NYCIII. 1000 μL of the overnight culture (or unspent media control) was transferred into a sterile, 24-well, tissue culture-treated polystyrene plate (Corning Costar #3524). The plate was incubated anaerobically at 37 °C for 24 hrs. Media in the wells was gently aspirated off from the side of the well, taking care not to disturb the biofilm on the bottom. Fresh anaerobic NYCIII media was added to the wells, and the plate was transferred to a 37 °C, 5% CO_2_ incubator for 24 hrs.

### Crystal violet staining

Quantification of biofilms by crystal violet staining was performed as previously described (O’Toole, 2011), with some slight modifications. To remove media and planktonic bacteria from the wells before crystal violet staining, each well was gently rinsed twice with 1000 μL of PBS. The well bottoms were then stained with 1000 μL of crystal violet for 15 minutes. The wells were again washed twice with PBS and air dried for 20 minutes. To elute the crystal violet, 500 μL of 70% ethanol was added to the wells and incubated for 15 minutes. The ethanol was then transferred to a 96-well plate, and the amount of crystal violet was quantified by reading absorbance at 590 nm.

### Sutures

Two monofilament and two braided suture types were selected for the study based on an informal survey of preferences by UC San Diego Maternal-Fetal Medicine faculty. The most common suture choices for transvaginal cerclage included polypropylene monofilament (Prolene^®^ 8825G), nylon monofilament (Ethilon^®^ D4573), braided polyester (Mersilene^®^ D1643), and braided polybutylate-coated polyester (Ethibond^®^ 825G). Sutures in size 2 were purchased (Ethicon, Cincinnati Ohio).

### Growth of Gardnerella biofilms on monofilament and braided sutures

**Figure 1:**
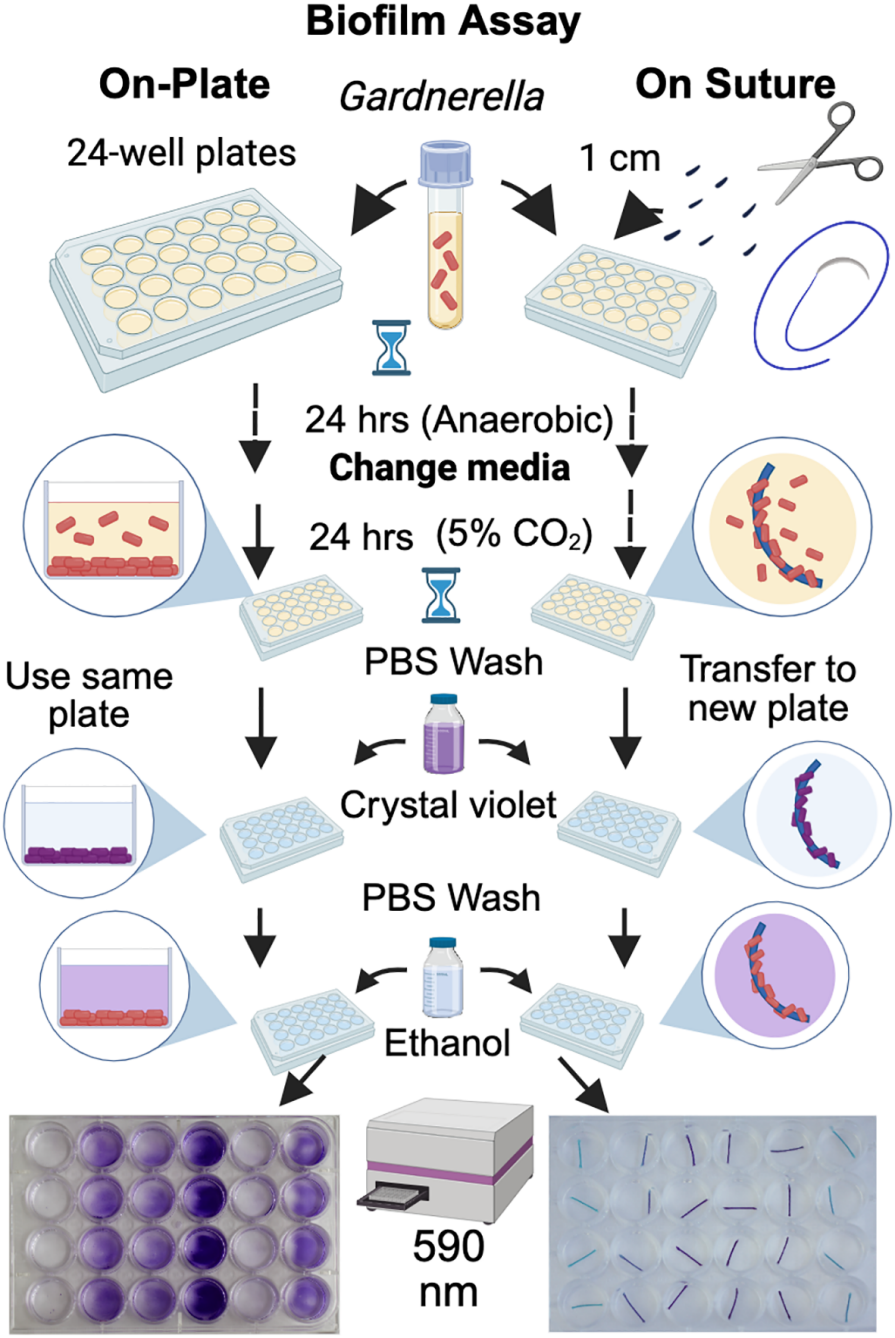
*Gardnerella* biofilm assay on tissue-culture treated polystyrene plates or surgical sutures made of different materials. The “On-Suture” assay is a modification of previously reported biofilm assays, where crystal violet staining is used to measure bacterial biomass in the mature biofilms (Gilbert et al., 2019). **Figure 1** was created in BioRender. Lewis, A. (2026) https://BioRender.com/kyh2j7p.

The canonical way to study *Gardnerella* biofilms in the laboratory has been to observe and measure their accumulation on tissue culture-treated polystyrene plates. However, it is not clear if this is a clinically relevant solid surface for biofilms. Here we present a new quantitative assay to measure *Gardnerella* biofilm formation on suture materials, incubated in 24-well plates with NYCIII bacterial culture media (**Figure 1**). Briefly, in a biosafety cabinet, sutures were removed using sterile stainless steel forceps from their packaging and cut to 1 cm pieces in petri dishes using a razor blade. In each experiment, three technical replicates of each suture type were included. Sutures were placed in 24-well tissue culture-treated plates. Preparation of the bacterial inoculum, addition to the 24-well plate, and incubation for the first 24 hrs were performed identically to the biofilm plate assay described above. Prior studies have used 10% CO_2_ conditions to grow *Gardnerella* biofilms (Castro and Cerca, 2015; Castro et al., 2019; Rosca et al., 2022a). Here, *Gardnerella* JCP8151B biofilms were initially grown anaerobically for 24 hours at 37 °C. At 24 hrs, sutures were transferred with sterile forceps into fresh anaerobic NYCIII media. Sutures moved to 5% CO_2_ for the second half of the incubation demonstrated significantly higher biomass (p = 0.0498) compared to biofilms grown entirely in the anaerobic chamber (**Supplementary Figure 1**). Therefore, all subsequent assays used 5% CO_2_ conditions at 37 °C after the media change at 24 hrs.

### Crystal violet staining and elution from sutures

After the biofilm growth period, sutures were transferred into a new 24-well plate using sterile forceps. Each suture was stained with crystal violet in the plate wells as described above. To account for the possibility that sutures may absorb some of the crystal violet in the absence of bacterial exposure, uninoculated (blank suture controls) were included in each assay.

### Statistical analysis

All statistical analyses were performed using Graphpad Prism version 10.4.2 (534).

One-way ANOVA was performed to compare between experimental groups. When the ANOVA indicated a statistically significant effect (alpha 0.05), *post hoc* pairwise comparisons were conducted using the Šidák correction to adjust for multiple comparisons between groups. As recommended by Graphpad Prism, Šidák was chosen because it controls the family-wise error rate (multiple comparisons between groups). P < 0.05 was considered statistically significant. **** P<0.0001.

## Results

**Figure 2:**
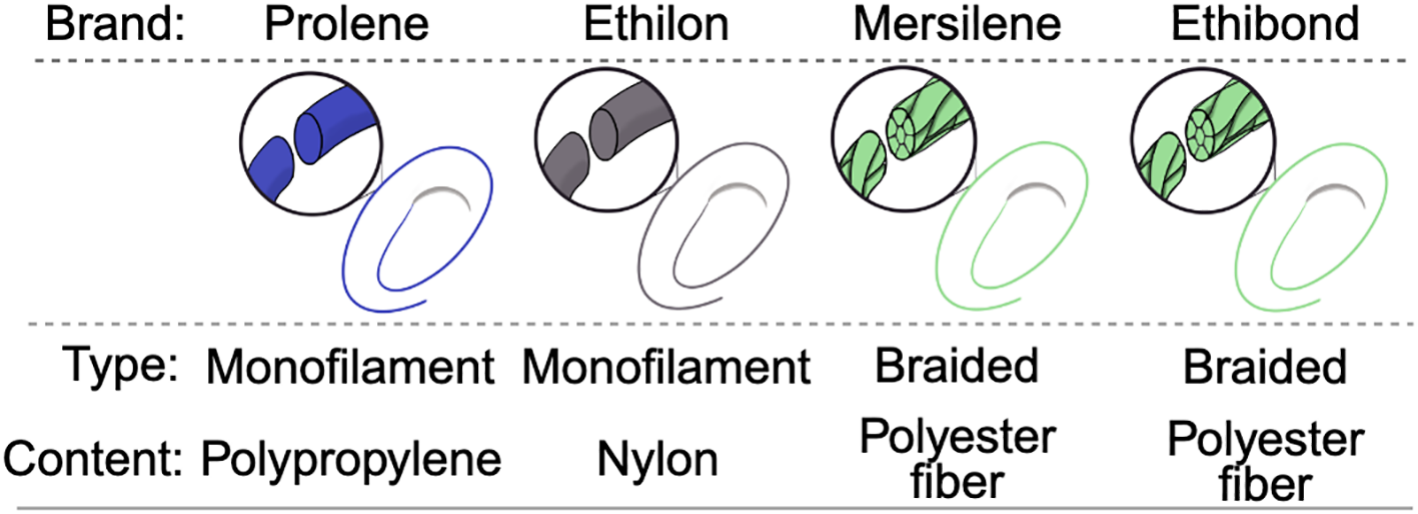
Schematic depiction of braided and monofilament sutures.

**Figure 3:**
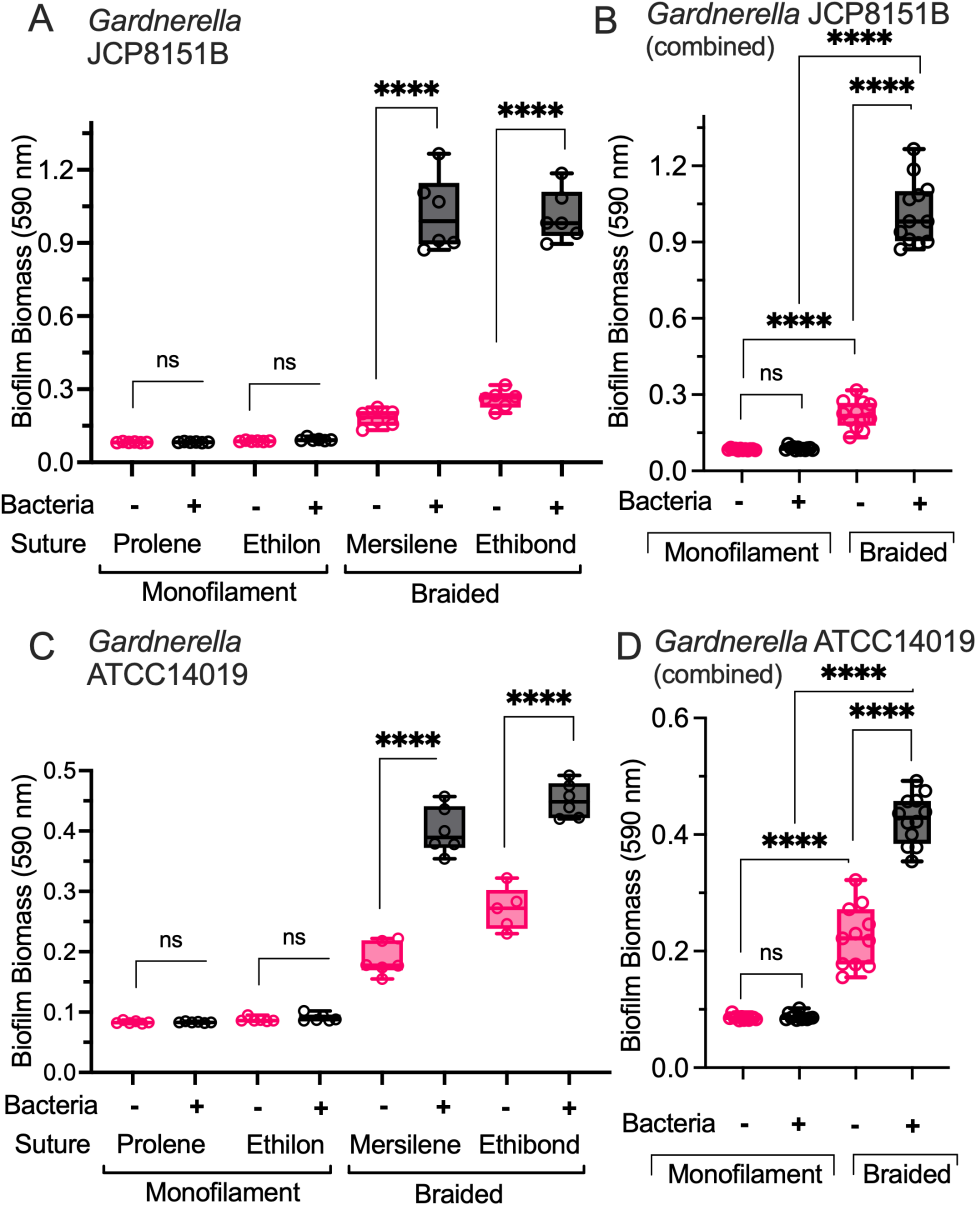
*Gardnerella* forms biofilm on braided, but not on monofilament sutures. Four suture types [Prolene; Ethilon; Mersiline & Ethibond] were tested with two *Gardnerella* strains, JCP8151B **(A, B)** and ATCC14019 **(C, D)**. Biomass of the resulting biofilms was measured by crystal violet staining (absorbance at 590 nm). **(A, C)** Experimental groups with bacteria were compared to a set of control sutures incubated without bacteria, and the two groups were stained in parallel. Two independent experiments were conducted for each suture type and control group, and three technical replicates per group were included in each experiment. **(B, D)** The same data for suture types were combined and reduced to two categories, “monofilament” and “braided.” One-way ANOVA with Šidák used to perform selected pairwise comparisons were performed, and applying the Šidák correction for multiple testing. ****P<0.0001.

*Gardnerella* biofilm formation on braided and monofilament sutures was measured using the new “On-Suture” biofilm assay conditions shown in **Figure 1**. Comparison of biomass accumulation on the suture is shown in **Figure 2**. Compared to negative controls without bacteria, the polypropylene monofilament (Prolene) and nylon monofilament (Ethilon) sutures yielded no statistically distinguishable biofilm growth on their surfaces when incubated with *Gardnerella* JCP8151B (**Figure 3A**) (e.g. mean Ethilon absorbance 0.087 to 0.094 in the absence and presence of bacteria respectively). In contrast, the *Gardnerella* JCP8151B strain produced a striking accumulation of biomass on the braided (Mersilene and Ethibond) sutures—far in excess over background (**Figure 3A**) (e.g. mean Mersilene absorbance of 0.182 to 1.021 in the absence and presence of bacteria, respectively). A somewhat higher background absorbance signal (i.e. staining in the absence of bacteria) was observed for braided compared to monofilament sutures (**Figure 3B**).

This finding was not limited to a single *Gardnerella* strain. In addition to the JCP8151B strain, the *Gardnerella* type strain ATCC14019 also formed heavy biofilm growth on the braided but not the monofilament sutures (**Figures 3C, D**). In total, five *Gardnerella* strains (JCP8151B, JCP8070, JCP7672, JCP8108, and ATCC14019) demonstrated biofilm-forming capabilities on braided sutures (**Supplementary Figure 2**). In each case, the apparent biofilm mass on braided sutures was greater than the “no bacteria” negative. Different *Gardnerella* strains displayed varying amounts of biofilm formation on the sutures.

## Discussion

Here, a classic biofilm assay was adapted to test whether *Gardnerella*, a bacterium known to overgrow in BV and believed to be the principal component of epithelial biofilms that form during this condition, was capable of forming robust biofilms on sutures. Braided sutures (Mersilene & Ethibond) proved highly amenable to biofilm growth compared to monofilament sutures (Prolene and Ethilon) when tested in parallel. Five strains of *Gardnerella* were able to form biofilms on braided sutures, underscoring the general conclusion that braided sutures are more permissive than monofilament sutures for the growth of *Gardnerella* biofilms.

These laboratory findings are concordant with an earlier prospective study (Kindinger et al., 2016) which showed a clear shift toward a BV-like microbiota in individuals receiving braided, but not monofilament, sutures. The data are also consistent with earlier laboratory findings that *Gardnerella* form robust biofilms on laboratory materials, and that these biofilms can incorporate other BV bacteria (Christensen et al., [[NoYear]]; Fredricks et al., 2005; Castro et al., 2019; Castro et al., 2020; Rosca et al., 2022b). The retrospective chart review of n=671 cerclage cases reported in Kindinger, 2016 found that braided sutures were associated with nonviable pregnancy and preterm birth (Kindinger et al., 2016). However, a large prospective randomized trial did not see differences in preterm birth, but instead found significantly higher rates of chorioamnionitis and maternal sepsis in participants receiving braided versus monofilament sutures (Hodgetts Morton et al., 2022). Additional studies are needed to further clarify the outcomes, as well as defining the microbiome structure longitudinally, which was not attempted in the larger trial.

On the surface, another study by Battarbee et al. testing both braided and monofilament sutures appears to address a similar question (Battarbee et al., 2019). However, the only comparison made was between “thin” versus “thick” sutures. The “thin” sutures used in the study included both braided and monofilament sutures, and there were no direct comparisons made between monofilament and braided sutures (Battarbee et al., 2019).

Braided sutures have been implicated in several types of post-surgical infections at different body sites. This finding has been shown in environments densely populated with bacteria like the oral cavity (Rodríguez Zorrilla et al. (2020)) and the gastrointestinal tract (Kathju et al., 2014), as well as in deeper tissues like knee joints (Swearingen et al., 2016). Given these findings, the use of braided sutures in the vaginal lumen, an environment inherently populated by bacteria, warrants caution. Notably, bacteria associated with BV have also been isolated from biofilms on contraceptive intrauterine devices and intravaginal rings (Hardy et al., 2017; Ádám et al., 2018).

The laboratory results presented here are not immediately applicable to clinical practices, but they nevertheless have direct clinical implications. Cervical cerclage with exposed suture in the vagina (McDonald cerclage) may create unique opportunities for potential pathogens and thbe development of a nonoptimal microbiome. Additionally, preoperative preparation of the vaginal canal and perioperative antibiotics could themselves potentially disrupt the normal vaginal microbiota. While necessary to prevent postsurgical infections, further studies are needed to test if these procedures create opportunities for potential pathogens to gain a foothold in the vagina after cerclage placement, or inhibit healthy lactobacilli from repopulating after surgery. In our study, *in vitro* exposure of the suture to bacteria is limited to 24 hours. This differs from the clinical use of sutures in cervical cerclage, where the suture is retained in the uterine cervix for weeks or months.

These experiments provide a straightforward illustration of one possible explanation for shifts in the vaginal microbiota after cerclage placement. The data suggests that *Gardnerella* can form biofilms on braided sutures placed in the cervix. These biofilms may be responsible for seeding the observed shift toward a BV-like microbiome (Kindinger et al., 2016), and may contribute to poor outcomes after cerclage placement. Additional prospective longitudinal studies are needed to directly compare the effects of braided and monofilament sutures on the vaginal microbiome in relation to pregnancy outcomes. Factors such as suture type and presurgical antimicrobial preparation could also be important for other types of gynecologic surgical procedures in the transvaginal setting, and could also have implications for other medical devices placed into the genital tract, such as pessaries and vaginal rings.

Strengths of this study include the relevance of *Gardnerella*, which is found in large quantities in BV biofilms and is capable of enabling the virulence of other potential pathogens in multiple settings (Fredricks et al., 2005; Swidsinski et al., 2005; Gilbert et al., 2017; Agarwal et al., 2020; Gilbert et al., 2021). Anaerobic growth conditions used in the initial biofilm growth may be a more relevant representation of BV, considering the site of suture placement. Using suture surfaces to objectively compare biofilms grown *in vitro* is also a strength and may be more relevant clinically than biofilm growth evaluated on other abiotic surfaces.

One limitation of this study is its focus on only one taxonomic group of bacteria, *Gardnerella*. Although it is a prominent BV member, *Gardnerella* is known to form biofilms in BV alongside other members like *Fannyhessea* (previously Atopobium) *vaginae (*Castro et al., 2019; Castro et al., 2020*).* As noted earlier, additional studies are needed to put these findings into clinical context.

While the mechanistic underpinnings of the shift to a BV-like microbiome are not yet clear, BV remains a misunderstood amplifier of gynecologic and obstetric complications that should be carefully considered in the setting of cervical cerclage and suture choice.

## Author‘s note

This work was presented in part at the 11th Annual Microbiome & Probiotic R&D & Business Collaboration Forum held by Global Engage in San Diego, California, USA from October 19 to 20, 2023.

## Data Availability

Requests to access the datasets should be directed to a1lewis@health.ucsd.edu.
