# Supplementary material for: Gardnerella biofilm formation in vitro is facilitated by braided sutures: implications for cervical cerclage
Source: Front Cell Infect Microbiol. 2026 Mar 24;16:1763531. doi: 10.3389/fcimb.2026.1763531 (PMC13055511; doi:10.3389/fcimb.2026.1763531)
Supplement: Supplementary file 1 [file Table1.docx]

**Supplementary 1. Anaerobic preparation of the initial biofilm inoculum yields biofilms with higher biomass.** Biofilm formation on polystyrene plates was investigated after 48 hrs with washing and a media change at 24 hrs. A two tailed t-test was used to examine statistically significant differences between the experimental groups (p = 0.0498*).


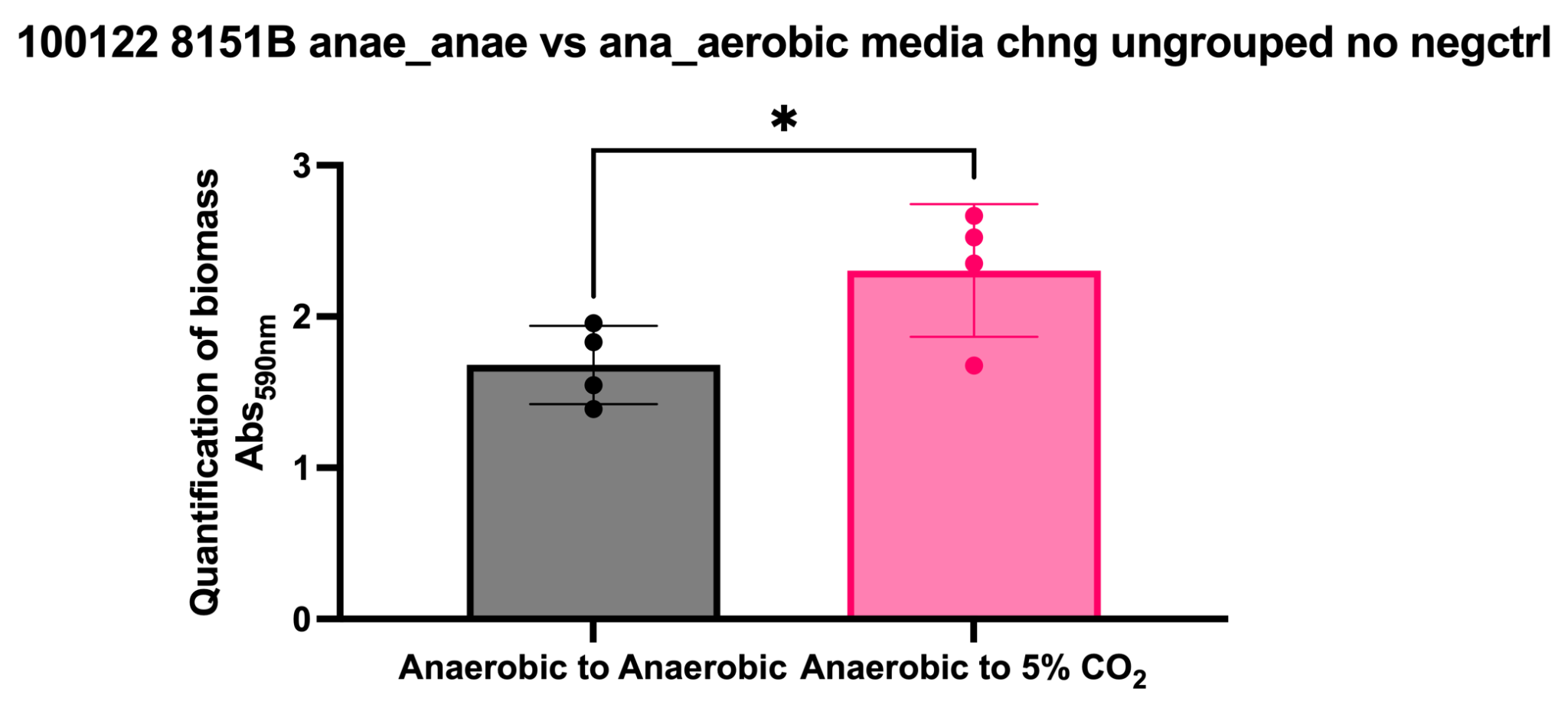


**Supplementary 2. Gardnerella biofilm mass on polystyrene plate wells does not necessarily correlate to approximate biofilm mass on braided polyester sutures.**


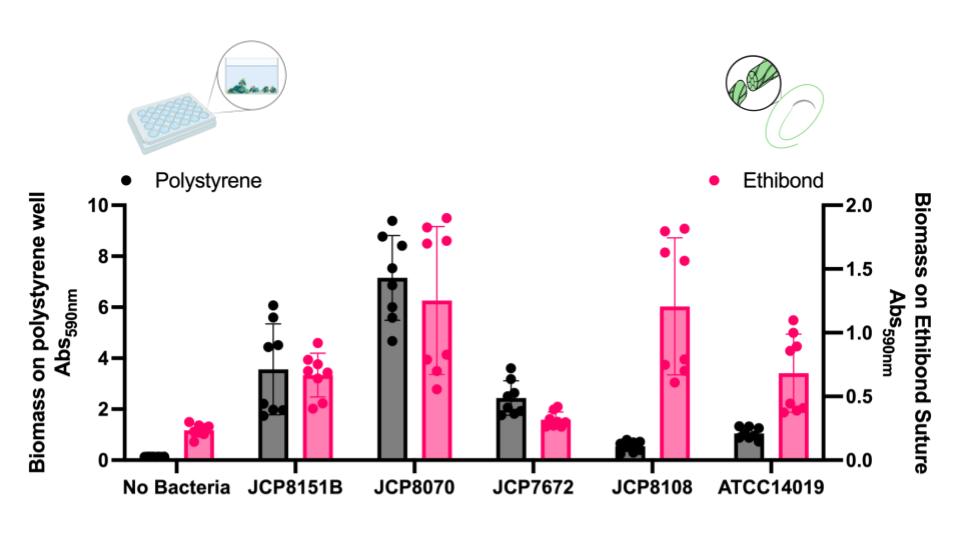


**Supplementary Table 1. Gardnerella incubation conditions under anaerobic and 5% CO_2_ yield adherent biofilms.**

| Characteristic | Incubation Condition |
| --- | --- |
| Starting OD_600_ | 0.1 |
| Plate | 24-well tissue-culture treated polystyrene |
| Bacteria volume added | 1000 μL |
| Incubation total time | 48 hours |
| 0-24h oxygen conditions | Anaerobic |
| 24-48h oxygen conditions | 5% CO_2_ |
| Media change | Every 24 hours |
| Wash liquid | PBS(-/-) |
| Wash repeats | 2 times |
| Staining reagent | 0.1% Crystal Violet |
| Elution reagent | 500 uL of 70% EtOH |
